# Farther Account of the Case of a Negro Woman Who Performed the Cæsarean Operation on Herself

**Published:** 1786

**Authors:** 


### XIII. *Farther Account of the Caſe of a Negro Woman who performed the Cæſarean Operation on Herſelf.*

SINCE the former account * of this caſe made its appearance, we have been favoured with ſome curious additional information concerning it, by Dr. David Morton, a very reſpectable phyſician at Kingſton, in Jamaica, who is at preſent in London, and who is mentioned by Dr. Brodbelt, in his letter to Mr. Cawley, as having had the care of the patient.—From this gentleman we learn that the

* Vol. VI. p. 372.

patient, who was well formed and in her fourth pregnancy, made the incision, not with a sharp knife, as Dr. Brodbelt had been informed, but with a blunt butcher's knife, the point of which was broken off. The wound which was much larger than would have been necessary for the extraction of the fœtus, was sewed up by a negro midwife, who, contenting herself with reducing the intestines, left the placenta in the uterus.

The patient made the incision about eight o'clock at night, and Dr. Morton who saw her about three hours after, found her without pulse, and lying on a mat on the floor. He immediately cut open the stitches, extracted the placenta through the wound, and after carefully removing, from the surface of the intestines, several bits of straw, and dirt, that had been left adhering to them by the midwife, sewed up the wound in a proper manner, and covered it with the usual dressings.

Dr. Morton saw her again at five the next morning, and then the pulse could be distinctly felt at the wrist, and the patient was able to speak. The child died on the fifth day, of tetanus, a disease which proves extremely fatal to new-born infants in tropical climates; but the mother, inftead of being attacked with dyfentery, and dying on the eleventh day, as was reported to Dr. Brodbelt, went on gradually recovering, and the wound was completely healed in five weeks. This happened in the year 1769, and the patient foon after her recovery became the property of Mr. Philips, of the parifh of St. Thomas in the Eaft. Dr. Morton being defirous to learn the fequel of her hiftory, made application, for that purpofe, by letter, about five years ago, to the furgeon who has the care of the negroes on Mr. Philips's eftate, and from him he learned that fhe was then in good health, and had lately been delivered, at the full term, of a living child—Such are the outlines of this very curious cafe, of which it is to be hoped Dr. Morton will, at his leifure, favour the public with a more circumftantial account.

